# Open Trial of Vitamin B12 Nasal Drops in Adults With Myalgic Encephalomyelitis/Chronic Fatigue Syndrome: Comparison of Responders and Non-Responders

**DOI:** 10.3389/fphar.2019.01102

**Published:** 2019-09-20

**Authors:** C (Linda) MC van Campen, Klaas Riepma, Frans C. Visser

**Affiliations:** ^1^Department of Cardiology, Stichting Cardiozorg, Hoofddorp, Netherlands; ^2^Department of Pharmacology, CureSupport, Valkenburg, Netherlands

**Keywords:** Sensewear™ armband, myalgic encephalomyelitis/chronic fatigue syndrome, vitamin B12 nasal drops, vitamin B12 serum concentration, RAND 36 questionnaire

## Abstract

**Introduction:** A recent study reported a favorable effect of vitamin B12 injections/oral folic acid support in myalgic encephalomyelitis/chronic fatigue syndrome (ME/CFS) patients. Recently, vitamin B12 nasal drops were developed as an alternative to the vitamin B12 injections. As no data are available on efficacy of this formulation, we studied vitamin B12 serum levels, the physical activity scale of the RAND-36, the number of steps on an activity meter, and the fatigue and concentration scales of the CIS20r questionnaires, before and after 3 months of treatment in ME/CFS patients.

**Methods and Results:** Fifty-one patients completed all measurements. Forty-four were female. Mean age was 42 years, and mean disease duration was 16 years. Median vitamin B12 levels before treatment were 328 (244–429) pmol/l, and 973 (476–1,476) pmol/l after treatment. Thirty-four patients reported a favorable response to treatment. In the non-responders, only a small but significant increase in vitamin B12 levels was observed. In contrast, in responders, the number of steps, the physical activity scale of the RAND-36, and the vitamin B12 serum levels increased significantly. The CIS20r fatigue scale decreased significantly, and the CIS20r concentration scale was unchanged.

**Conclusions:** Nasal drop vitamin B12 administration resulted in a significant increase in vitamin B12 serum levels and therefore may be effective. This pilot study suggest that the nasal drops may be used as an alternative to injections because two thirds of ME/CFS patients reported a positive effect, accompanied by an increased number of steps, improvement of the RAND-36 physical functioning scale and the CIS20r fatigue scale, and a significant increase in serum vitamin B12 levels.

## Introduction

Myalgic encephalomyelitis/chronic fatigue syndrome (ME/CFS) is a severe and often disabling chronic disease (Fukuda et al., 1994; Carruthers et al., 2011; IOM, 2015). Although the exact pathophysiological mechanism of ME/CFS is unknown, multiple studies have shown that inflammatory, metabolic, and immunological abnormalities play an important role (IOM, 2015). As the exact pathophysiology is unknown, there are no curative treatments available. Therefore, treatments aiming to improve part of the symptomatology are currently in use (Teitelbaum et al., 2006; Maric et al., 2014; Castro-Marrero et al., 2015; Witham et al., 2015; Campagnolo et al., 2017).

In 2015, Regland et al. (Regland et al., 2015) were the first to report a favorable effect of vitamin B12 injections/oral folic acid support on the patients’ global impression score and the fibro fatigue scale in ME/CFS patients (Zachrisson et al., 2002). In that study, factors associated with a more positive response included a higher dose of vitamin B12 injections, a higher injection frequency, a higher dose of folic acid support, and a longer treatment duration. Furthermore, the long term treatment (up to 20 years) proved to be safe. A limitation of that study was that patients were included only if they had first experienced a favorable response to vitamin B12 intramuscular injections.

Recently, vitamin B12 nasal drops were introduced as an alternative to the vitamin B12 injections and oral supplementation (hydroxocobalamin 5,000 mcg produced by CureSupport). Thus far, no data are available on the effectiveness of vitamin B12 nasal drops on serum levels or on the patient response to this treatment.

Therefore, the aim of this study was twofold: 1) to assess vitamin B12 serum levels in ME/CFS patients before and after use of high dose vitamin B12 nasal drops and 2) to compare the changes in function before and after starting therapy, using an activity meter, the physical functioning score of the Rand 36 questionnaire, and both the fatigue and concentration scales of CIS20r questionnaire.

## Patients, Material, and Methods

Between 2015 and 2016, 66 patients with ME/CFS were studied (Fukuda et al., 1994; Carruthers et al., 2011). Based on their symptoms, the diagnosis of CFS according to the Fukuda Criteria (Fukuda et al., 1994) and ME according to the new International Consensus Criteria (Carruthers et al., 2011) was established. To fulfil the Fukuda (Fukuda et al., 1994) criteria for CFS, patients had to have chronic fatigue complaints for at least 6 months and to have at least four out of eight cardinal criteria. In all patients, diagnoses, which could explain the fatigue, were ruled out. No important co-morbidities were present. Disease duration and presence or absence of fibromyalgia were noted. The diagnoses of fibromyalgia – if not diagnosed previously – was made using the validated modified ACR criteria (Ferrari and Russell, 2013; Wolfe et al., 2016). Additionally, treatment with thyroid hormones and analgesics was scored.

The usual care of the study participants included a detailed clinical history, physical examination, vitamin B12 serum levels (pmol/l), and ECG and echocardiography. All participants completed the Rand 36 (Dutch version) (Viehoff et al., 2008) and CIS20r questionnaire (Vercoulen et al., 1994). Furthermore, patients wore a Sensewear^TM^ armband for 5 days before starting treatment. Patients were instructed to use high dose vitamin B12 nasal drops (5,000 mcg one nasal drop in each nostril, twice per week). After a treatment period of 3 months, to test the effectiveness of treatment as part of usual clinical care, vitamin B12 levels, activity meter, and questionnaires were repeated.

We obtained the physical functioning sub-scale from the Rand 36 questionnaire, along with the fatigue and concentrations scores from the CIS20r questionnaire. Higher scores on the SF-36 and lower scores on the CIS20 indicate improvement of symptoms. We measured the mean number of steps per day using the Sensewear^TM^ armband. For vitamin B12 serum concentrations, total vitamin B12 levels as standardized measurements in laboratories were used. The accuracy of vitamin B12 levels ranged up to 1476 pmol/l. Values above 1476 pmol/l were reported as >1476 pmol/l. No additional diluting of samples was done. Patient outcome scale: after the treatment period, patients were asked on a five-point scale whether they perceived an effect of the treatment. Scores were as follows: 1: substantially deteriorated, 2: mildly deteriorated, 3: no effect, 4: mildly improved, and 5: substantially improved.

If patients reported improvement (from mild to substantial), they were regarded as responders; the other patients were defined as non-responders.

The trial was conducted in accordance with the Declaration of Helsinki (1996 revision) and under the principals of good clinical practice, as laid out in the International Conference on Harmonization document Good Clinical Practice Consolidated Guideline.

All patients gave written informed consent to be treated, and the study using clinical data for research was approved by the medical ethical committee of the Slotervaart Hospital.

### Data Analysis

Data were analyzed using SPSS 21. The distribution of values was tested by the one-sample KS test, and data are presented as the mean +/− SD or as median and IQR, where appropriate. For the comparison between responders and non-responders, the unpaired t test was used. For comparison within the groups before and after treatment, we used the paired t test. A p-value < 0.05 was considered significantly different.

## Results

Sixty-six patients entered the study. Five patients were excluded because of high vitamin B12 serum levels before the start of treatment (related to previous use of vitamin B12 tablets or vitamin B12 injections). Two patients were unable to use the drops due to technical reasons and one had sesame oil allergy (the basis of the hydroxocobalamin nasal drops). Two patients did not complete the treatment period due to side effects, and five more did not complete the posttreatment measurements.

A total of 51 individuals with ME/CFS (44 female) completed all measurements before and after the treatment period. Twenty-eight met criteria for fibromyalgia (FM). The mean age was 42 ± 12 years, and the mean disease duration was 15.7 ± 9.3 years. For the entire group, the median vitamin B12 level before treatment was 328 (range, 244–429) pmol/l, and 973 (range, 476–1476) pmol/l after treatment. The physical functioning score of the RAND-36 was 40 ± 32% pretreatment and 46 ± 19% posttreatment. The CIS20r fatigue scale was 51 ± 6 pretreatment and 50 ± 6 posttreatment, and the CIS20r concentration scales were both 28 ± 6 pretreatment and posttreatment. Thirty-four of the 51 patients reported a favorable response to the treatment, and 17 did not.

**Table 1** shows the baseline results of the non-responder and responder groups. There was a slight imbalance in the male: female -ratio, with significantly more males in the non-responder group (p < 0.02). There was no difference between groups for age, disease duration, or the number of patients having FM. Non-responders had a significantly higher physical functioning scale of the RAND-36 pretreatment than responders (51 ± 21 vs. 34 ± 18; p < 0.01). Non-responders had a significantly higher mean number of steps per day pretreatment compared to non-responders (4,246 ± 2,888 vs. 3,480 ± 2,393; p < 0.005). There was no difference between groups in the proportion receiving thyroid hormone replacement therapy and/or use of analgesics.

**Table 1:** Baseline characteristics compared between responders and non-responders.

	Responder (n = 34)	Non-Responder (n = 17)	p-value
Male/Female (n)	2/32	5/12	<0.02
Age (years)	40 (12)	46 (13)	ns
Fibromyalgia (n)	19	9	ns
Disease duration (years)	15.2 (8.8)	16.7 (10.3)	ns
Number of steps pre	3480 (2393)	4246 (2888)	<0.01
Number of steps post	5773 (2137)	6024 (2304)	<0.05
RAND-36 physical activity pre (%)	34 (18)	51 (21)	<0.01
RAND-36 physical activity post (%)	41 (18)	53 (20)	ns
CIS20r fatigue scale pre	52 (5)	49 (7)	ns
CIS20r fatigue scale post	50 (6)	49 (7)	ns
CIS20r concentration pre	29 (4)	26 (9)	ns
CIS20r concentration post	29 (4)	26 (9)	ns
B12 levels pre (pmol/l)	379 (294;437)	265 (228;360)	<0.05
B12 levels post (pmol/l)	1445 (975;1476)	395 (356;452)	<0.0001
Thyroid medication (n)	2	1	ns
Analgesics (n)	10	3	ns

N, number; pre, pre-treatment; post, post-treatment; B12 levels presented as median and IQR; ns, not significant.

**Table 2** shows the results of the measurements before and after treatment with vitamin B12 nasal drops. In the responder group, the number of steps, the physical activity scale of the RAND-36, and the vitamin B12 serum levels increased significantly. The CIS20r fatigue scale decreased significantly (indicating improvement), although the CIS20r concentration scale was unchanged. In contrast, the only significant change among non-responders was in the vitamin B12 levels.

**Table 2:** Clinical measurement characteristics compared before and after treatment.

	Pretreatment	Posttreatment	p-value
Responders (n = 34)			
Number of steps	3480 (2393)	4246 (2888)	<0.0001
RAND-36 physical activity scale (%)	34 (18)	41 (18)	<0.0001
CIS20r fatigue scale	52 (5)	50 (6)	<0.01
CIS20r concentration scale	29 (4)	29 (4)	ns
Vitamin B12 levels (pmol/l)	379 (289;442)	1445 (972;1476)	<0.0001
**Non-Responders (n = 17)**			
Number of steps	5773 (2137)	6024 (2304)	ns
RAND-36 physical activity scale (%)	51 (21)	53 (20)	ns
CIS20r fatigue scale	49 (7)	49 (7)	ns
CIS20r concentration scale	26 (9)	26 (9)	ns
Vitamin B12 levels (pmol/l)	265 (228;373)	395 (354;473)	<0.0001

N, number; B12 levels presented as median and IQR; ns, not significant.

**Figure 1:**
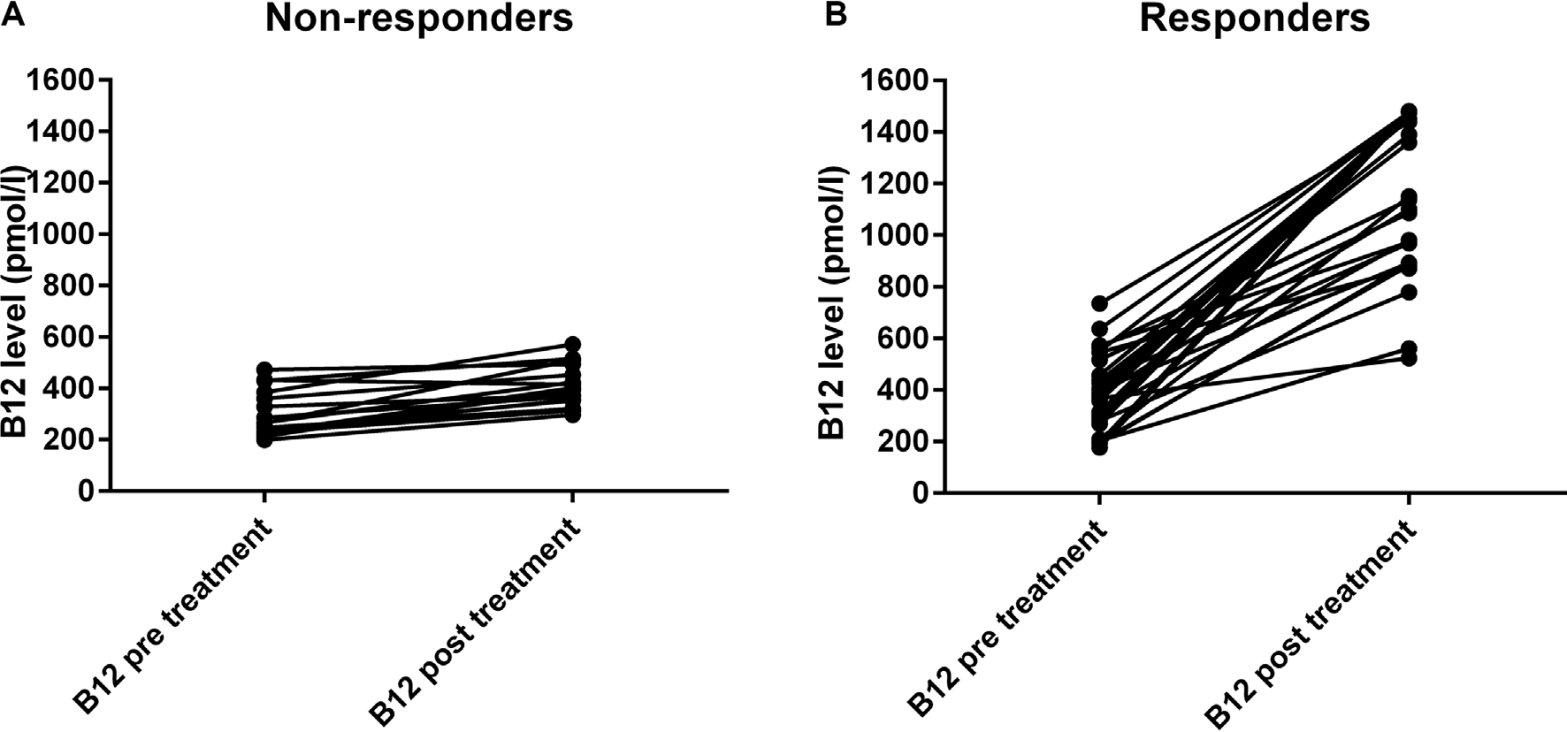
Serum vitamin B12 levels (pmol/l) before and after 3 months treatment. The panel **(A)** shows the non-responders (n = 17), and the panel **(B)** shows the responders (n = 34).

**Figure 1** shows the individual vitamin B12 serum levels before and after treatment for the non-responders and responders. The increase in vitamin B12 levels in non-responders was significant but much lower than the increase in levels among the responders. The median increase of vitamin B12 levels in the non-responder group was 97 pmol/l (IQR: 78, 173) and in the responder group 934 pmol/l (IQR: 571, 1130).

## Discussion

To the best of our knowledge, this is the first clinical study in ME/CFS patients describing a novel administration route of vitamin B12. We are not aware of studies describing the use of intranasal vitamin B12 in other diseases. The study showed that the administration of 10.000 ugr of hydroxocobalamin nasal drops twice weekly was associated with a significant increase in vitamin B12 serum levels over the course of 3 months, starting from a median of 328 pmol/l (range 244–429) to 973 pmol/l (range 473–1,476). Among 51 ME/CFS patients treated in this fashion, 2/3 reported improvement, accompanied by an increase in number of steps per day, an increase in the physical functioning scale of the RAND-36, and an improvement in the CIS20r fatigue scale. In social media, improvement in mental concentration is often mentioned as one of the main effects to be expected. However, this could not be confirmed using the CIS20r concentration scale.

Those who reported no improvement in global well-being had a minimal change in vitamin B12 levels, no significant improvement in the physical functioning scale of the RAND-36, no significant increase in number of steps, and no significant improvement in the CIS20r fatigue scale. The most plausible explanation is that the non-responders used incorrect administration of the nasal drops. Another possibility is the following: after uptake, vitamin B12 is slowly bound to serum vitamin B12 proteins (Neale, 1990) and the free vitamin B12 is rapidly excreted in urine (Sokoloff et al., 1952). Interestingly, the excretion is similar between patients with and without pernicious anemia. The percent excretion is increased with increasing doses of administration (Sokoloff et al., 1952). This suggests that the non-responders with a minor increase in vitamin B12 serum concentration have either a low vitamin B12 protein saturation or a very rapid urinary excretion compared to the responders. This hypothesis needs further study.

The mechanism by which vitamin B12 administration exerts its positive effect on ME/CFS patients is unknown. Regland et al. suggested a difference in the individual MTHFR gene variation (Regland et al., 2015). The authors also suggested that the megalin receptor in the choroid plexus may play a role.

In contrast to the aforementioned study (Regland et al., 2015), our study found no imbalance in fibromyalgia, treatment with analgesics, or treatment with thyroid hormone replacement therapy.

Importantly, we did not preselect patients based on a positive response to previous vitamin B12 injections, leading to more generalizable results in our study. **Table 1** shows that the number of steps and the physical function scale of the RAND-36 were significantly higher in the non-responders. This suggests that non-responders were less severely affected by ME/CFS at baseline, and that the main positive effect of nasal B12 administration occurs in more severely affected patients. These hypotheses will need to be explored in a randomized study.

Limited data are available on the use of vitamins for symptomatic relieve in ME/CFS patients. Two studies used multivitamins for symptom relieve (Brouwers et al., 2002; Maric et al., 2014). In an observational study, Maric et al. found a significant decrease in fatigue, sleep disorders, autonomic nerve system symptoms, headaches, and a decrease in subjective feeling of infection after the use of a multivitamin preparation. In contrast, in a randomized placebo controlled study, Brouwers et al. found no differences between placebo and active treatment. Additionally, one randomized placebo controlled study found no effect of vitamin D suppletion on CFS related symptoms (Witham et al., 2015).

### Limitations

The present study does not describe long term efficacy and/or safety. Regland et al. (Regland et al., 2015) described long term safety, even up to a period of 20 years. They describe a difference in response with higher doses of vitamin B12 injections and oral folic acid, more frequent injections, and longer treatment duration, but this was not supported by higher vitamin B12 serum results or objective measures other than patient reported outcomes. Second, the present study is quite small with respect to sample size, and therefore, inclusion bias may have been present. Third, no other vitamin levels (e.g., folic acid) were studied. This needs to be addressed in future studies.

Based on our results, a randomized placebo controlled study of intranasal B12 is warranted. Furthermore, a crossover study of intranasal vitamin B12 versus vitamin B12 injections is needed. In addition, one or both of these trials could include investigation of whether adding a high dose of folic acid would improve outcomes.

## Conclusion

Two thirds of an unselected group of ME/CFS patients treated for 3 months with vitamin B12 nasal drops had a favorable outcome in objective measures and subjective patient reported outcome, which were associated with a significant increase in serum vitamin B12 levels.

## Data Availability

The raw data supporting the conclusions of this manuscript will be made available by the authors, without undue reservation, to any qualified researcher.

## Ethics Statement

The trial was conducted in accordance with the Declaration of Helsinki (1996 revision) and under the principals of good clinical practice, as laid out in the International Conference on Harmonization document Good Clinical Practice Consolidated Guideline. All patients gave written informed consent to be treated, and the study using clinical data for research was approved by the medical ethical committee of the Slotervaart Hospital.

## Author Contributions

CC, KR, and FV conceived the study. CC and FV collected the data. CC performed the primary data analysis, and FV and KR performed secondary data analyses. All authors were involved in the drafting and review of the manuscript.

## Funding

This research did not receive any specific grant from funding agencies in the public, commercial, or not-for-profit sectors.

## Conflict of Interest Statement

KR is employed by CureSupport.

The remaining authors declare that the research was conducted in the absence of any commercial or financial relationships that could be construed as a potential conflict of interest.
